# Top coal drawing law for an extra thick coal seam under the single round group drawing method

**DOI:** 10.1038/s41598-024-65831-6

**Published:** 2024-07-03

**Authors:** Weidong Pan, Zhining Zhao, Xinyuan Li, Yongxin Xu, Kunming Zhang

**Affiliations:** 1 Engineering Research Center for Green and Intelligent Mining of Thick Coal Seam, Ministry of Education, Beijing, 100083 China; 2 School of Energy and Mining, China University of Mining and Technology, Beijing, 100083 China; 3 Consulting Centre of China National Coal Association, Beijing, 100013 China; 4 Department of Energy and Mineral Engineering, Pennsylvania State University, University Park, PA 16802 USA

**Keywords:** Coal, Civil engineering

## Abstract

To solve the problems of poor top coal drawing, lagging collapse, and difficulty in parallel operation of mining and drawing in extra-thick coal seams, considering the 8222 working face of the Tashan Mine as the engineering background, we first investigate the basic problems of fully mechanized top coal drawing mining in extra-thick coal seams using the single-round group drawing method (GDM). We then analyze the drawing law of top coal with different thicknesses under the single-round GDM from the aspects of top coal recovery (TCR) and drawing efficiency, coal loss mechanism, and the relation between TCR and gangue content (GC), providing a basis for determining the process parameters of GDM. Results indicate that as the top coal thickness increases, the number of drawing openings considerably influences drawing efficiency and top coal loss. And there is a notable thickness effect of the number of drawing openings on the top coal loss. There is a quantitative relationship among TCR, cumulative GC (CGC) and instantaneous GC (IGC), and CGC and TCR can be predicted based on the IGC. Consequently, theoretical analysis and numerical simulation results indicate that the optimal IGC threshold at the coal drawing openings between 31.2 and 40%. Through optimizing the coal drawing method and strictly controlling the IGC at the coal drawing openings on-site, the measured working face TCR increased from 75.25 to 90.12%, and CGC was controlled at approximately 9%. Meanwhile, the average coordination efficiency of mining and drawing time reaches 68.2%, effectively ensuring the construction of a coal mine with an annual output of 15 million tons.

## Introduction

In the central and western regions of China, such as Shanxi, Inner Mongolia, and Xinjiang, there are abundant reserves of extra-thick coal seams (thickness: ≥ 8 m), and fully mechanized drawing is the primary mining method for such coal seams^1–3^. The two major challenges of long drawing times and frequent top coal arching during mining in such coal seams severely restrict the improvement of coal drawing efficiency in working faces. As we all know, the coal drawing method not only has a great influence on the TCR and GC in working faces, but also affects the total drawing speed and the completion of the regular cycle, which in turn affects the high yield and high efficiency of the working face. Therefore, choosing or designing appropriate coal drawing methods based on on-site conditions is the main means of improving drawing efficiency and TCR^4,5^.

**Figure 1:**
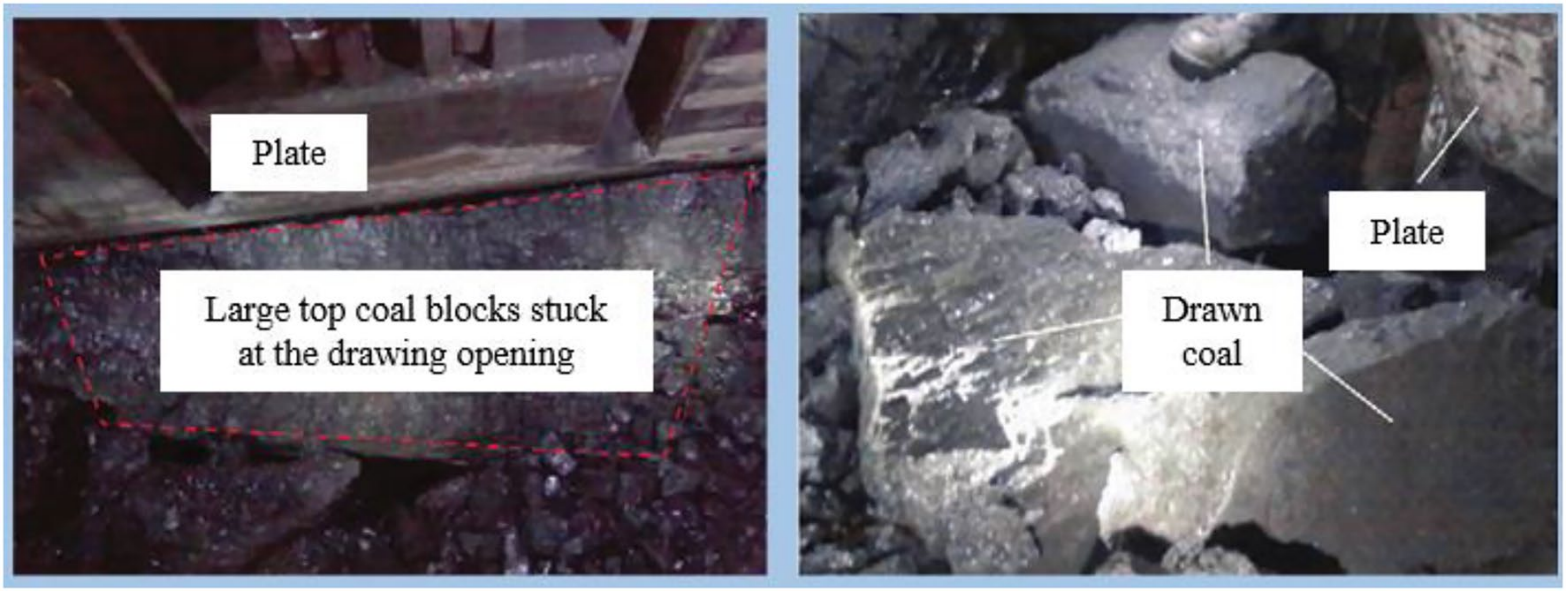
Insufficiently broken top coal in an extra-thick coal seam.

At present, several scholars at home and abroad have conducted extensive and effective research on the theory and optimization of coal drawing methods in fully mechanized caving mining of thick coal seams to reduce residual coal between supports and improve TCR^6–12^. From the current research, it can be seen that the research results of coal drawing theory and methods mostly focus on thick coal seams and single-support coal drawing. However, with the continuous increase in the first mining thickness in fully mechanized mining, conventional drawing methods are becoming increasingly difficult to meet the needs of high-intensity mining in current working faces, seriously restricting the improvement of production capacity in working faces as well as the high-yield and efficient construction of mines. And the limited area of single-opening coal drawing, poor top coal drawing, and easy arching of drawing openings cause several problems, such as low drawing efficiency, low TCR rate, and high mixed GC^13^. In particular, in some thick coal seam fully mechanized working faces, such as the 3–5# coal seam in the Tashan Mine, Datong Mining Area, the coal seam thickness ranges from 8 to 20 m, and the coal seam hardness coefficient is relatively high. Particularly, the Proctor hardness coefficient of the Jurassic coal seam can reach 2.9–4.4^14,15^. For such working faces with high coal drawing height and poor top coal drawing performance, large blocks of coal gangue are prone to arching and blocking coal drawing openings during coal drawing^16–19^ (Fig. 1), thereby increasing the drawing time and reducing the working face production efficiency. Meanwhile, frequent arching and arch-breaking can intensify the coal–rock interface disturbance during coal drawing, causing problems, such as gangue intrusion into the coal flow and early closure of the drawing openings. The difficulty in smoothly drawing large blocks of top coal also restricts the improvement of TCR in working faces. In addition, subject to the level of automation and intelligence in the fully mechanized working face, manual operation has always been the main coal drawing method. The design and selection of coal drawing methods must fully consider the operability of workers. In this context, the single-round sequence and single-round interval drawing method based on single-opening coal drawing have become mainstream. When the top coal drawing property is good and the drawing height is not high, this type of coal drawing method can adapt well to the needs of mining in working faces. As the top coal thickness increases, the traditional single-opening coal drawing method becomes increasingly difficult to meet the requirements of high-intensity mining in thick coal seams, and TCR is difficult to ensure, which seriously restricts the improvement of production efficiency in working faces as well as the high-yield and efficient construction of mines. To improve the drawing efficiency of such working faces, the coal drawing method must inevitably transition from single-opening drawing to multi-opening drawing, that is, indirectly increasing the spatial size of the drawing opening through the method of simultaneous coal drawing from multiple openings, thereby improving the coal drawing efficiency^13,20–26^. With the continuous development and progress of mining technology, the fully mechanized working face is gradually transitioning from mechanization to automation and intelligence^27^. Moreover, the main equipment performance of fully mechanized working faces based on coal mining machines, scraper conveyors, and fully mechanized drawing supports continues to improve, including support control capabilities and scraper conveyor conveying capabilities, offering possibilities for the application of group coal drawing methods.

Based on this, “Key Technologies and Demonstration of Intelligent Fully Mechanized Drawing Mining for Ten Million Ton Extra-Thick Coal Seams” (2018YFC0604500), a national key research and development plan project in the 13th Five Year Plan, proposes the use of the group drawing method (GDM) to achieve high-yield and efficient development of a 20-m extra-thick coal seam fully mechanized working face. On this basis, we use theoretical analysis, numerical simulation methods, and on-site measurements to investigate the drawing law of different thicknesses of top coal under single-round GDM from the aspects of TCR and drawing efficiency, coal loss mechanism, and the relation between TCR and GC, providing a basis for determining the process parameters of GDM. In addition, we provide ideas and technical guidelines for the efficient and high recovery development of such coal seams in China.

## Theory of top coal drawing using group drawing method

### Top coal drawing body and coal–rock interface

**Figure 2:**
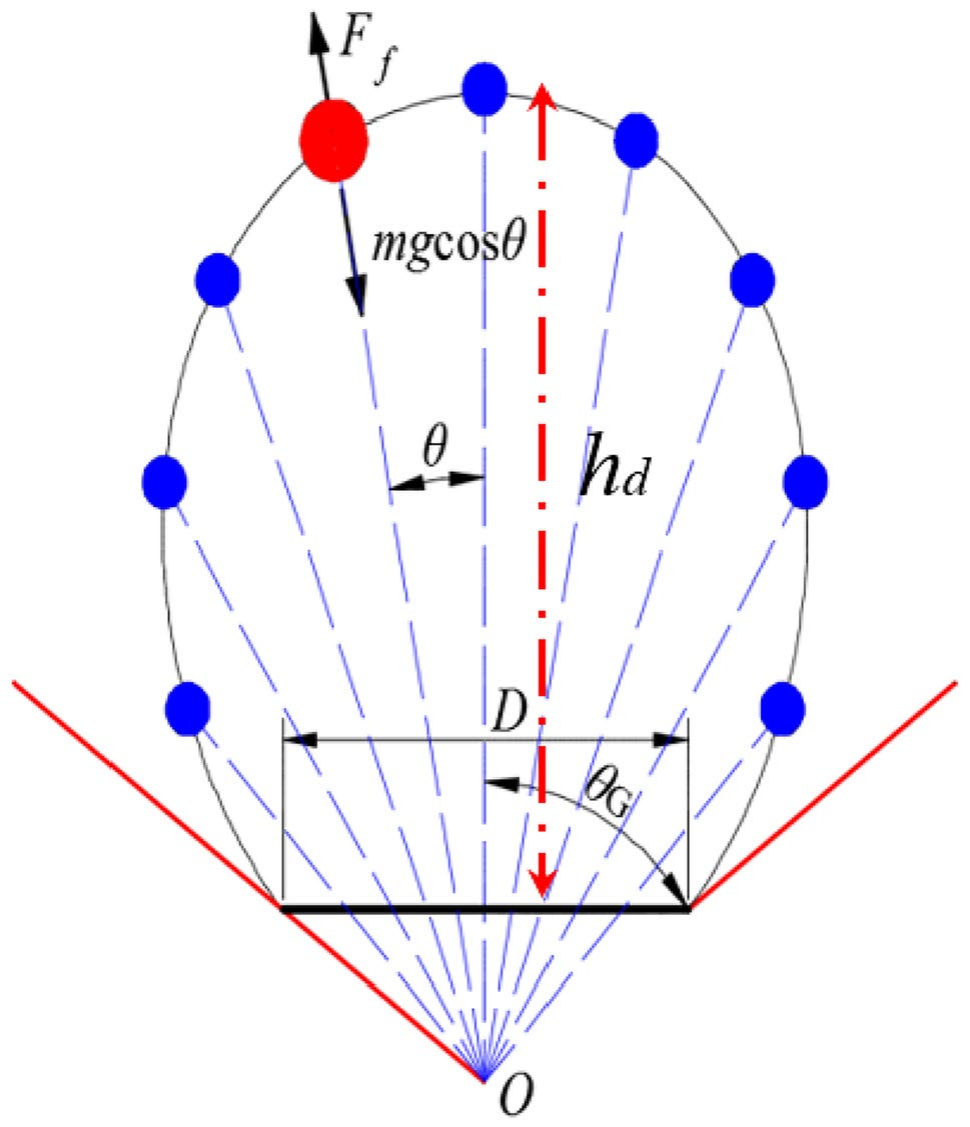
Schematic of the B–R model.

Because the development of the top coal drawing body toward the working face layout is unaffected by the fully mechanized drawing support, the boundary of the top coal drawing body can be described using the Bergmark-Roos (B–R) model^6,26^ (Fig. 2). According to the original assumption of the B–R model^28^, the distance between the top coal and the pole on the top coal drawing body boundary can be expressed as follows:

1 $$r_{B} (\theta ) = \frac{{\sin \theta - \sin \theta_{G} }}{2}gt^{2} + r_{dd}$$

where *θ* denotes the angle between the movement trace of the top coal and the horizontal direction, $^\circ$(i.e., polar angle), *θ*_*G*_ denotes the critical transport angle of the top coal, $^\circ$, *g* is the gravitational acceleration, m/s^2^, *t* denotes the time required for the top coal on the drawing body boundary to be transported to the drawing opening, s, *nL* denotes the total length of the drawing opening, m, and *r*_*dd*_ denotes the distance between pole *O* and the plane where the drawing opening is located under the angle *θ*, m.

Taking the highest point to be located at *θ* = π/2 for the drawing body, the drawing height *h*_*d*_ can be expressed as follows:

2 $$h_{d} = \frac{{1 - \sin \theta_{G} }}{2}gt^{2}$$

By combining Eqs. (1) and (2), the boundary equation of the drawing body represented by the drawing height *h*_*d*_ can be obtained as follows:

3 $$r_{{\text{B}}} (\theta ) = \frac{{h_{d} (\sin \theta - \sin \theta_{G} )}}{{1 - \sin \theta_{G} }} + \frac{{nL\tan \theta_{G} }}{2\sin \theta }$$

**Figure 3:**
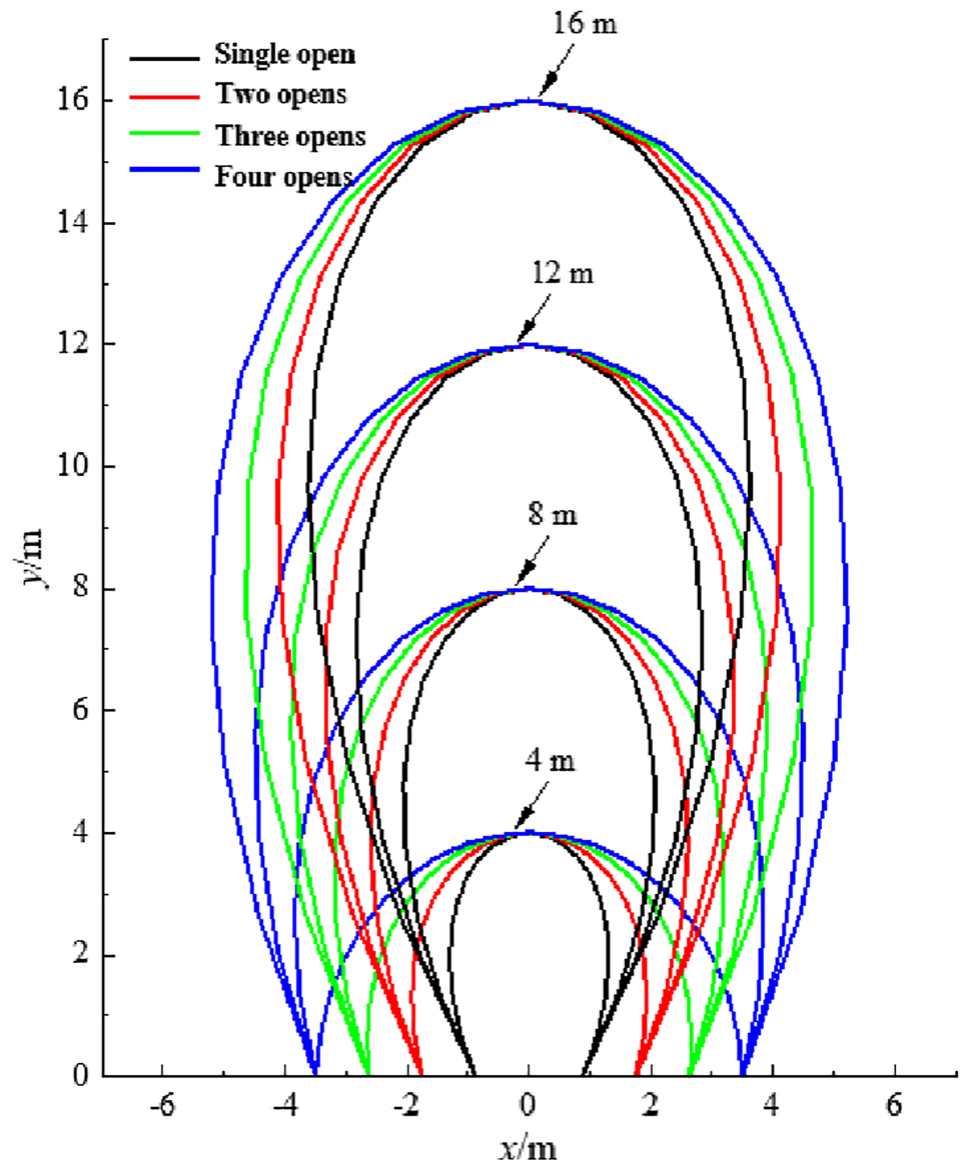
Development process of the top coal drawing body.

Depict the development process and morphological characteristics of top coal drawing bodies with different thicknesses in the working face layout direction according to Eq. (3) (Fig. 3). From the figure, when the drawing height is low, the top coal drawing body shows a “hemispherical defect” shape. However, as the coal drawing height increases, the top coal drawing body gradually develops into an “ellipsoidal defect” shape with a wider top and narrower bottom. The higher the coal drawing height, the more prominent the “ellipsoidal defect” feature becomes.

During the top coal drawing body development, the coal–rock interface also migrates and evolves, and the migration process of the coal–rock block at the coal–rock interface is still subject to the assumption of the B–R model. The coal–rock interface with an initial height of *h*_*r*_ gradually migrates and evolves with increasing coal drawing height *h*_*d*_, and its equation can be expressed as follows:

4 $$\begin{array}{*{20}c} {r_{{\text{b}}} (\theta ) = \frac{{nL\tan \theta_{G} + 2h_{r} }}{2\sin \theta } - \frac{{h_{d} (\sin \theta - \sin \theta_{G} )}}{{1 - \sin \theta_{G} }}} \\ \end{array}$$

### Instantaneous gangue content and cumulative gangue content

**Figure 4:**
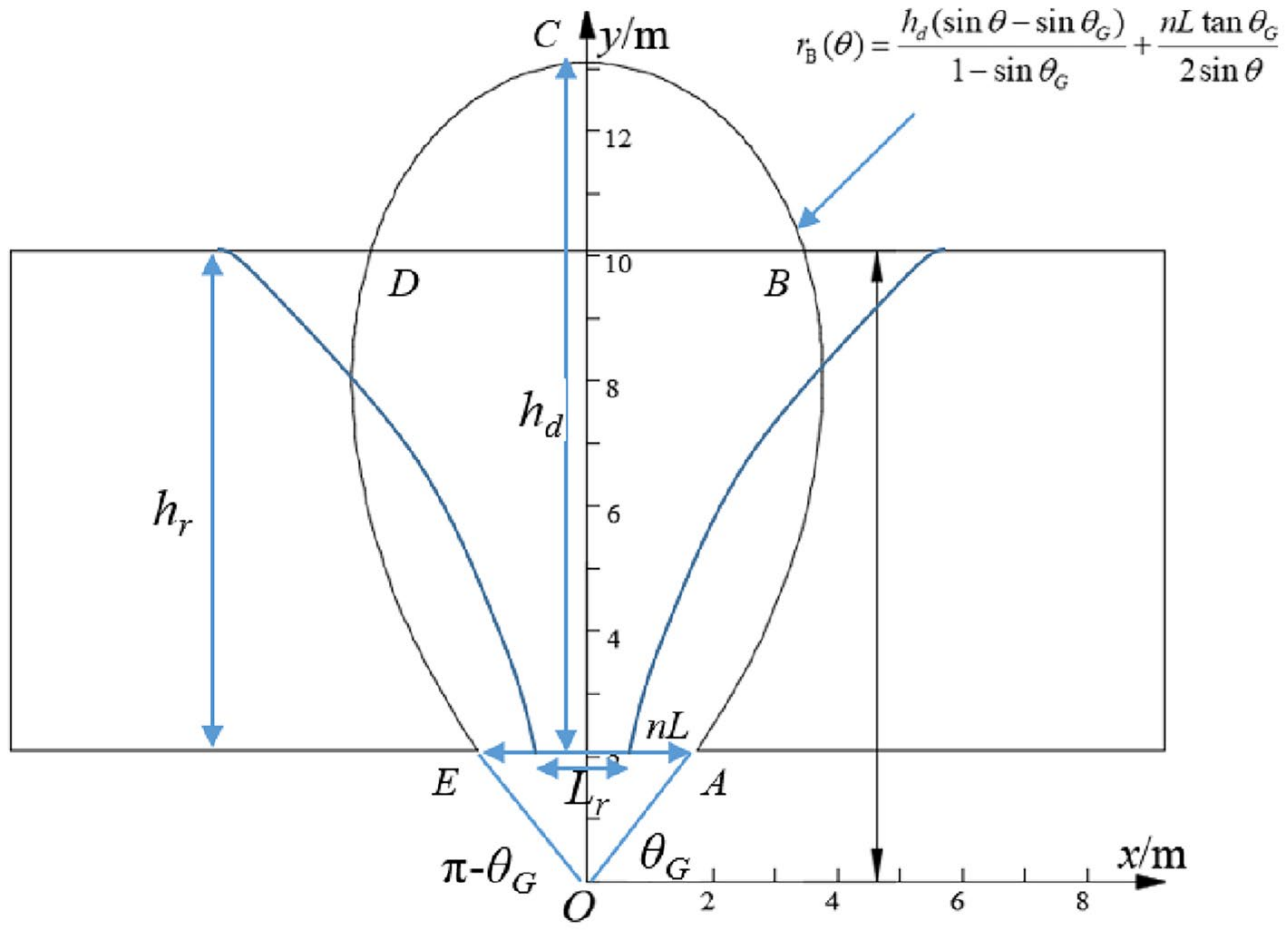
Calculation model of instantaneous and cumulative gangue contents under excessive coal drawing condition.

Establish a calculation model for the gangue content under an excessive coal drawing condition (Fig. 4). In the figure, *O* denotes the pole (origin), A and E denote the two endpoints of the drawing opening, B and D denote the intersection points of the drawing body and initial coal–rock interface, C denotes the highest point of the drawing body, and *L*_*r*_ denotes the distance between the intersection points of the drawing open and coal–rock interface, m. As shown in the figure, under an excessive coal drawing condition, the vertical distance between the intersection points between the drawing body and the original coal–rock interface and pole *O* (i.e., mean point of B and D) satisfies the following:

5 $$h_{r} + \frac{{nL\tan \theta_{G} }}{2} = \left( {\frac{{h_{d} (\sin \theta - \sin \theta_{G} )}}{{1 - \sin \theta_{G} }} + \frac{{nL\tan \theta_{G} }}{2\sin \theta }} \right)\sin \theta$$

By solving Eq. (5), the polar angles *θ*_*B*_ and *θ*_*D*_ of the two intersection points between the drawing body and the original coal–rock interface can be obtained as follows:

6 $$\left\{ \begin{gathered} \theta_{B} = \arcsin \left( {\frac{{\sin \theta_{G} + k}}{2}} \right) \hfill \\ \theta_{D} = \pi - \arcsin \left( {\frac{{\sin \theta_{G} + k}}{2}} \right) \hfill \\ \end{gathered} \right.$$

where $$k = \sqrt {\sin^{2} \theta_{G} + \frac{{4h_{r} (1 - \sin \theta_{G} )}}{{h_{d} }}}$$.

From Eq. (6), under an excessive coal drawing condition, the theoretical equation of the coal–rock interface needs to consider the range of values of *θ*. When *θ*_*G*_ ∈  (*θ*_*G*_, *θ*_*B*_) ∪ (*θ*_*D*_, π − *θ*_*G*_), the coal–rock interface still conforms to the original equation, but when *θ*_*G*_ ∈ *(θ*_*B*_, *θ*_*D*_), the coal–rock interface is cut off by the drawing opening. Therefore, under an excessive coal drawing condition, the theoretical equation of the coal–rock interface can be expressed as follows:

7 $$r_{b} \left( \theta \right) = \left\{ \begin{gathered} \frac{{nL\tan \theta_{G} + 2h_{r} }}{2\sin \theta } - \frac{{h_{d} \left( {\sin \theta - \sin \theta_{G} } \right)}}{{1 - 2\sin \theta_{G} }},\;\;\theta \in \left( {\theta_{G} ,\theta_{B} } \right) \cup \left( {\theta_{D} ,\pi - \theta_{G} } \right) \hfill \\ \frac{{nL\tan \theta_{G} }}{2\sin \theta },\theta \in \left( {\theta_{B} ,\theta_{D} } \right) \hfill \\ \end{gathered} \right.$$

According to the above equation, the distance between the midpoint of the drawing opening and the pole can be expressed as follows:

8 $$\frac{{{\text{n}}L\tan \theta_{G} }}{2} = \left[ {\frac{{nL\tan \theta_{G} + 2h_{{\text{r}}} }}{2\sin \theta } - \frac{{h_{d} (\sin \theta - \sin \theta_{G} )}}{{1 - \sin \theta_{G} }}} \right]\sin \theta$$

Under an excessive coal drawing condition, the instantaneous GC (IGC), *w*_*i*_, at the drawing opening can be expressed as follows:

9 $$w_{i} = \frac{{L_{r} }}{nL} = \frac{{\tan \theta_{G} }}{\tan \theta }$$

By combining Eqs. (8) and (9), the IGC at the drawing opening at any drawing height *h*_*d*_ (*h*_*d*_ ≥ *h*_*r*_) can be obtained as follows:

10 $$w_{i} = \frac{{tan\;\theta_{G} \sqrt {4 - (\sin \theta_{G} + k)^{2} } }}{{sin\;\theta_{G} + k}}$$

According to the drawing body area, the cumulative GC (CGC), *w*_*a*_, under an excessive coal drawing condition can be obtained as follows:

11 $$w_{a} = \frac{{\rho_{r} S_{{_{BCD} }} }}{{\rho_{r} S_{{_{BCD} }} + \rho_{{_{c} }} \left( {S_{{_{ABCDE} }} - S_{{_{BCD} }} } \right)}}$$

where *ρ*_*r*_ denotes the gangue density, kg/m^3^, *ρ*_*c*_ denotes the top coal density, kg/m^3^, and *S* denotes the corresponding drawing body area, m^2^ (Fig. 4).

S_BCD_ and S_ABCDE_ can be obtained from the area formula in polar coordinates, as follows:

12 $$\left\{ {\begin{array}{*{20}c} {S_{{{\text{BCD}}}} = S_{{{\text{OBCD}}}} - S_{{\Delta {\text{OBD}}}} = \frac{1}{2}\int_{{\theta_{B} }}^{{\theta_{D} }} r_{B}^{2} \left( \theta \right)d\theta - r^{2} (\theta_{{\text{B}}} )\sin \theta_{{\text{B}}} \cos \theta_{{\text{B}}} } \\ {S_{{{\text{ABCDE}}}} = S_{{{\text{OABCDE}}}} - S_{{\Delta {\text{OAE}}}} = \frac{1}{2}\int_{{\theta_{G} }}^{{\pi - \theta_{G} }} r_{B}^{2} \left( \theta \right)d\theta - \frac{{{\mathfrak{n}}^{2} L^{2} \tan \theta_{G} }}{4}} \\ \end{array} } \right.$$

By substituting the boundary equation r_B_(θ) into Eq. (12) and simplifying, we obtain

13 $$\left\{ \begin{gathered} S_{BCD} = \frac{{a^{2} + 16ac\sin \theta_{B} + 8b^{2} }}{{8\tan \theta_{B} }} - \frac{{(a^{2} + 4ab + 2c^{2} )(2\theta_{B} - \pi )}}{4} - 2bc\ln \, \tan \left( {\frac{{\theta_{B} }}{2}} \right) \\ - \frac{{a^{2} \cos (3\theta_{B} )}}{{8\sin \theta_{B} }} - \left( {a\sin \theta_{B} + \frac{b}{{\sin \theta_{B} }} + c} \right)\left( {h_{r} + \frac{{nL\tan \theta_{G} }}{2}} \right)\cos \theta_{B} \\ S_{ABCDE} = \frac{{a^{2} + 16ac\sin \theta_{B} + 8b^{2} }}{{8\tan \theta_{G} }} - \frac{{(a^{2} + 4ab + 2c^{2} )(2\theta_{G} - \pi )}}{4} - 2bc\ln \, \tan \left( {\frac{{\theta_{G} }}{2}} \right) \\ - \frac{{a^{2} \cos (3\theta_{G} )}}{{8\sin \theta_{G} }} - \frac{{n^{2} L^{2} \tan \theta_{G} }}{4} \\ \end{gathered} \right.$$

Notably, *a*, *b*, and *c* are given by

14 $$\left\{ {\begin{array}{*{20}c} {a = \frac{{h_{d} }}{{1 - \sin \theta_{G} }}} \\ {b = \frac{{nL\tan \theta_{G} }}{2}} \\ {c = \frac{{h_{d} \sin \theta_{G} }}{{1 - \sin \theta_{G} }}} \\ \end{array} } \right.$$

**Figure 5:**
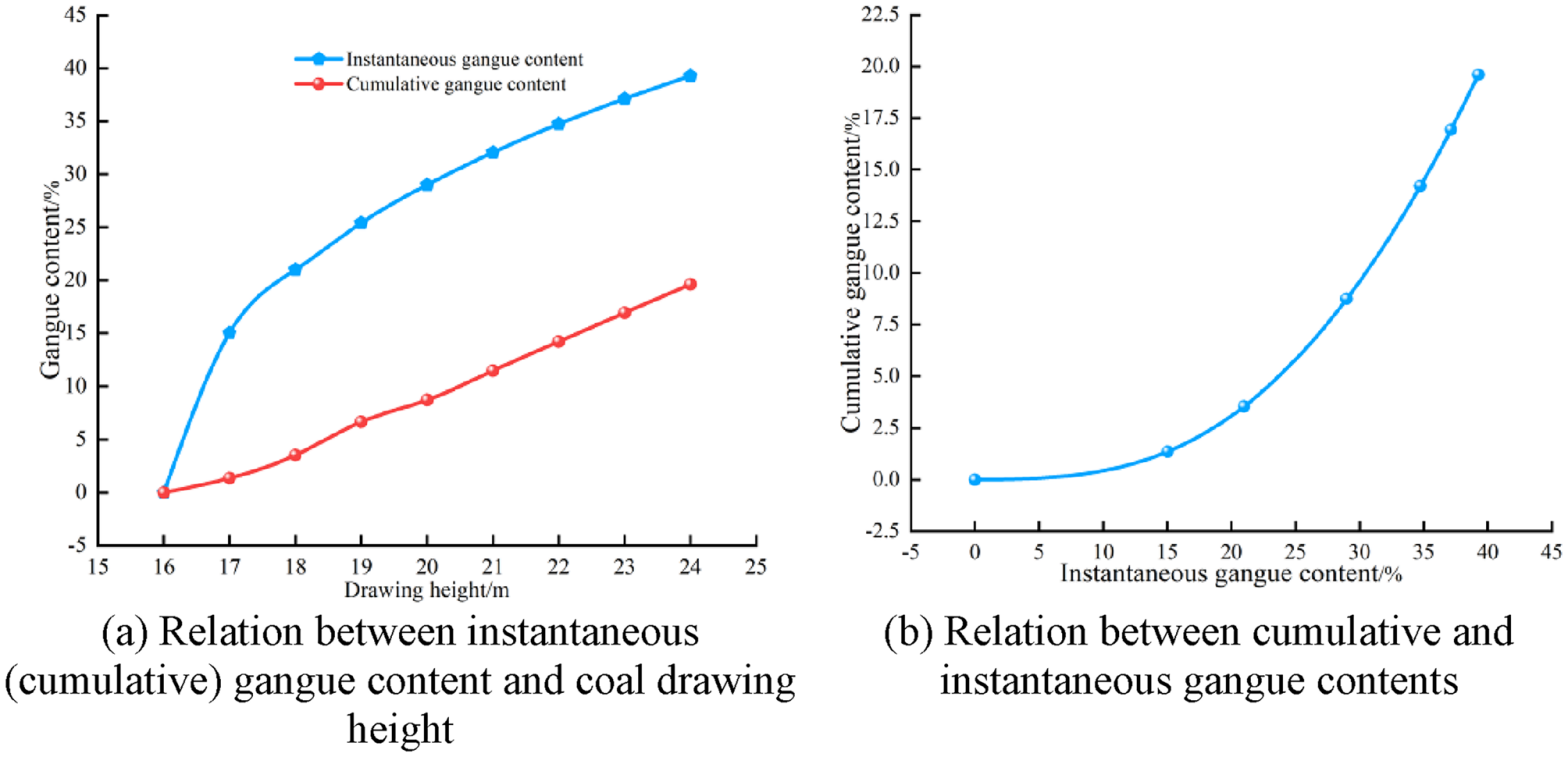
Relation between IGC and CGC during the initial coal drawing stage.

By substituting Eq. (13) into Eq. (11), the CGC during the initial coal drawing stage can be obtained. Considering the two-open group drawing under the condition of a top coal thickness of 16 m as an example, the relevant parameters *θ*_*G*_ = 40°, *h*_*r*_ = 16 m, *L* = 1.75 m, *n* = 2, *ρ*_*1*_ = 2560 kg/m^3^, and *ρ*_*2*_ = 1340 kg/m^3^ were used in the expressions of IGC and CGC; the relation curve between IGC and CGC under excessive coal drawing conditions is plotted in Fig. 5.

As shown in Fig. 5a, IGC and CGC gradually increase with coal drawing height during the initial coal drawing stage, but the growth rate of IGC showed a considerably slower trend. As shown in Fig. 5b, with an increase in IGC, CGC continues increasing with a gradually enhanced growth rate. This is because in the later stage of excessive coal drawing, as a large GC is drawn, the mass proportion of gangue in the coal gangue mixture gradually increases. In addition, the gangue density is generally much higher than that of coal; thus, the CGC can always increase at a large rate and eventually exceed the IGC. Previous studies have shown a statistical relation between TCR and CGC^29^. Therefore, it is possible to predict CGC and TCR using IGC.

## Top coal drawing law under group drawing method

### Model establishment and simulation plan

**Figure 6:**
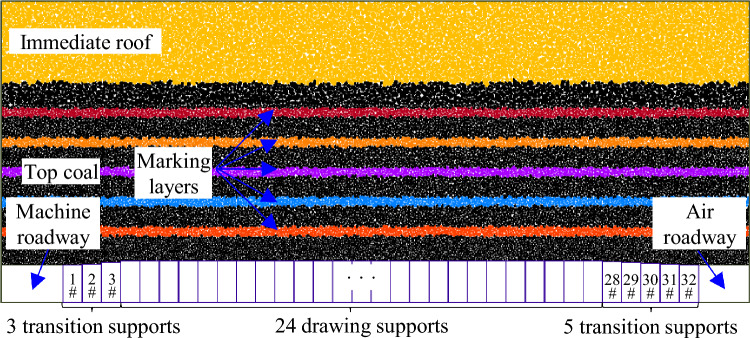
Numerical coal drawing test model for working face inclination (top coal thickness of 16 m).

**Figure 7:**
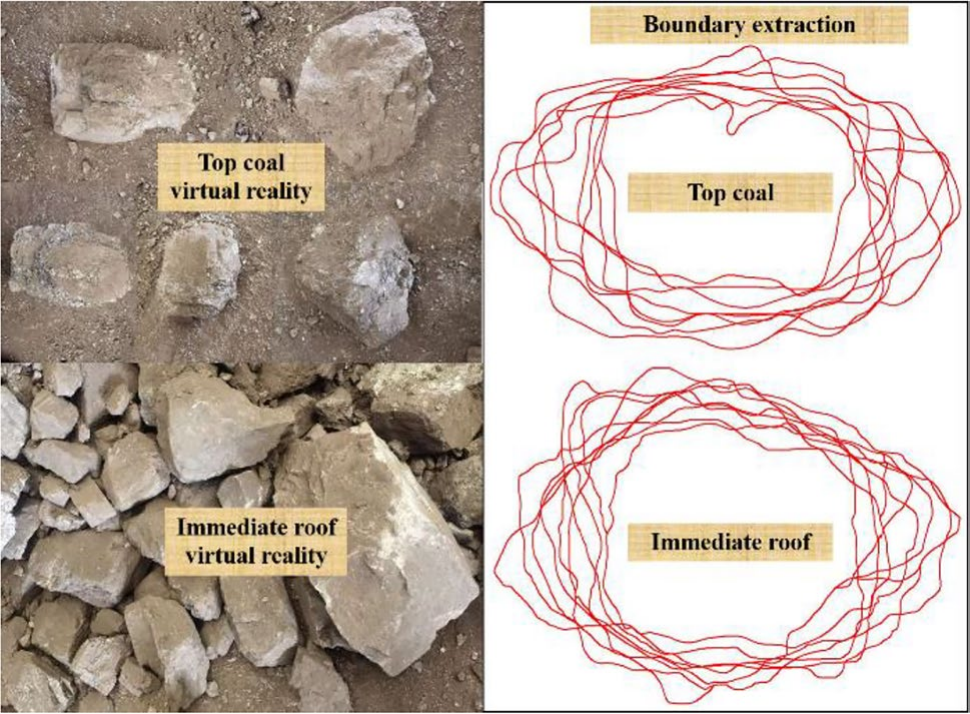
Actual morphology and boundary extraction of fractured coal and rock.

To investigate the drawing law of different top coal thicknesses under GDMs, based on the coal–rock occurrence conditions of the 8222 working face, a numerical drawing test model along the inclination of the working face using the particle flow code (PFC) software (https://www.itascacg.com/software/pfc) was established (Fig. 6). The entire model had a length and height of 66.2 and 27.4 m, respectively. The model mining height was 4.0 m, the top coal thickness was set 4 gradients (Table 1), and the immediate roof thickness was 7.4 m. The left side of the model was a machine roadway, with dimensions of 5.6 × 3.4 m^2^ (width × height), with an air roadway on the right, whose dimensions were 4.6 × 3.4 m^2^ (width × height). A total of 32 fully mechanized drawing supports were set between the model machine and air roadways, including 8 transition supports (no coal drawing) and 24 middle coal drawing supports (coal drawing). The center distance of the support was 1.75 m. To simulate coal and rock blocks more realistically, on-site images of the coal–rock blocks at the coal drawing support and sampling of the coal gangue blocks at the working face were collected to extract the boundary curves of the coal–rock blocks. After rotating and stacking the boundaries of the coal–rock blocks, we found that the top coal and rock blocks mostly had irregular polygonal shapes in two-dimensional space. Therefore, irregular blocks were used to simulate the broken top coal and immediate roof (Fig. 7). The simulated block size of the 8222 working face was set according to the measured block size^30^ (Table 2). Notably, the upper- and lower-top coals in Table 2 are within a 2-m range above and below the coal body. The other parts of the coal body are the middle–top coal. The degree distribution of the crushed immediate roof block is consistent with that of the upper-top coal. The displacement constraint boundary was applied on both sides and the bottom of the model, and the “wall” element in PFC was used to limit the coal–rock block movement. The top of the model was a free boundary without an external force, implying that the coal–rock block was only affected by its gravity and the gravity on the upper block.

**Table 1:** Coal drawing experiment scheme for different top coal thicknesses.

Scheme	Top coal thickness (m)	Number of drawing openings	Coal drawing order
1	4	2, 3, 4	Two openings: (4#, 5#)–(6#, 7#)–…–(27#, 28#) Three openings: (4#, 5#, 6#)–(7#, 8#, 9#)–…–(26#, 27#, 28#) Four openings: (4#, 5#, 6#, 7#)–(8#, 9#, 10#, 11#)–…–(25#, 26#, 27#, 28#)
2	8		
3	12		
4	16

**Table 2:** Model physical and contact mechanic parameters.

Materials	Density (kg·m^−3^)	Block area (size, cm^2^)	Equivalent block size (m)	Minimum size (cm^2^)	Elastic modulus (Pa)	Tangential normal stiffness ratio	Frictional coefficient
Top coal							
Upper	1360	N (0.12, 0.11^2^)	0.3–1.2	0.05	2.0 × 10^8^	2.54	0.5
Middle		N (0.06, 0.02^2^)	0.25–0.55	0.04			
Lower		N (0.035, 0.005^2^)	0.2–0.3	0.03			
Immediate roof	2560	N (0.12, 0.11^2^)	0.3–1.2	0.05	4.0 × 10^8^	2.58	0.7

**Figure 8:**
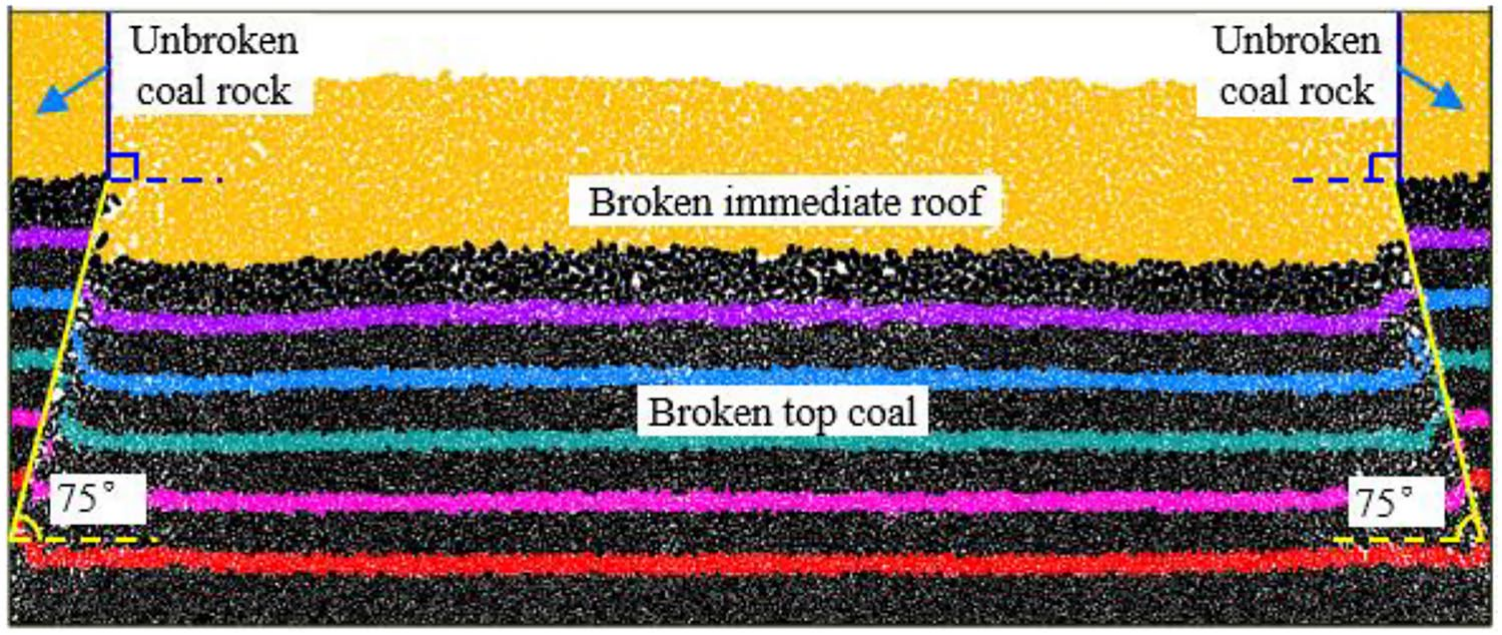
Top coal and immediate roof collapse and piling after support movement.

The collapse angle of the top coal in the 3–5 # coal seam is generally within 75°–85°. Because of the undeveloped cracks in the top coal above the two roadways of the working face and the influence of support materials, such as anchors, the collapse is relatively difficult. Therefore, the lower value of the top coal collapse angle above the two roadways is set to 75°, and the immediate roof collapse angle is set to 90°. Before drawing coal, we first removed the working face supports to simulate the collapse and accumulation of the top coal after the support removal and then applied displacement constraints to the top coal and immediate roof above the roadways to simulate the top coal and roof collapse (Fig. 8).

After the model stabilized, drawing tests using different drawing methods and top coal thicknesses were performed according to the sequence of support coal drawings shown in Table 1. During on-site coal drawing, workers generally determine the timing of closing the drawing by observing the volume proportion of gangue on the coal flow surface of the rear scraper conveyor. Therefore, coal workers actually use IGC as a judgment indicator to determine whether to terminate the coal drawing. In this case, the GC observed by workers is instantaneous and dynamic and will constantly change with an increase in drawn coal. When the coal drawing termination criterion of “closing the drawing opening upon seeing the gangue” is adopted in the working face, there is a worker’s reaction time and support action time during the process from observing the gangue appearance on the scraper conveyor to controlling the support to close the drawing opening. Inevitably, a certain amount of gangue will still be mixed in the drawn top coal. However, numerical simulation can monitor whether the gangue has reached the drawing opening at a very high frequency. If the “closing the drawing opening upon seeing the gangue” is still used as the termination condition for the coal drawing, there will be a considerable difference between the numerical simulation and the actual situation. Therefore, we considered a 10% IGC as the threshold to simulate the “closing the drawing opening upon seeing the gangue” coal drawing termination criterion in actual production.

After the experiment, the amount and time of coal drawing for different top coal thicknesses and the number of drawing openings were calculated, and the TCR and drawing efficiency were then calculated. Normally, the transition supports at both ends of the working face do not draw coal, and only a portion of the top coal above them can be drawn when coal is drawn through adjacent supports. In this case, the TCR statistics of the entire model cannot truly reflect the actual recovery situation of the top coal along the working face inclination. Therefore, statistical analysis was performed on the TCR situation in the actual drawing area of the model, and the TCR was determined. *η*_*dip*_ can be obtained using Eq. (15):

15 $$\eta_{{{\text{dip}}}} = \frac{{w_{d} }}{{w_{t} }} \times 100\%$$

where *w*_*d*_ denotes the mass of top coal drawn in the drawing area (the amount of top coal drawn from 4–28# supports), and *w*_*t*_ denotes the mass of all top coals in the drawing area of the initial model (the initial amount of top coal above 4–28# supports). As the name suggests, drawing efficiency is the mass of top coal drawn per unit time.

### Top coal recovery and drawing efficiency

**Figure 9:**
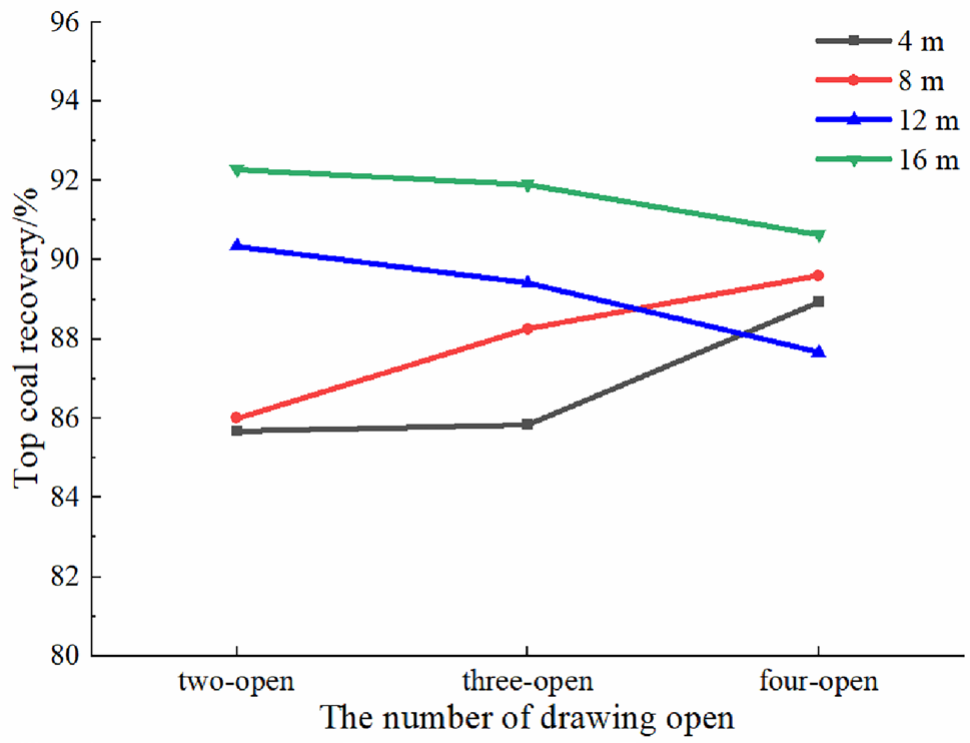
Top coal recovery in the drawing area using the single-round group drawing method.

To analyze the relation between the TCR and the number of drawing openings, the TCR of the entire drawing area was calculated (Fig. 9). For top coal thicknesses of 4 and 8 m, the TCR gradually increases with an increase in the number of drawing openings, whereas for top coal thicknesses of 12 and 16 m, the TCR gradually decreases with an increase in the number of drawing openings. Specifically, for the top coal thicknesses of 4 and 8 m, the TCR values of the two-opening GDM were 85.68% and 86.00%, respectively. Compared with the two-opening GDM, the TCR of the three (four)-opening GDM increased by 0.15% (3.25%) and 2.26% (3.60%), respectively. For the top coal thicknesses of 12 and 16 m, the TCR values of the two-opening GDM were 90.34% and 92.28%, respectively. Compared with the two-opening GDM, the TCR values of the three (four)-opening GDM decreased by 1.12% (3.51%) and 0.37% (1.65%), respectively. As a result, TCR depends on drawing time or efficiency as well as the number of drawing openings.

**Figure 10:**
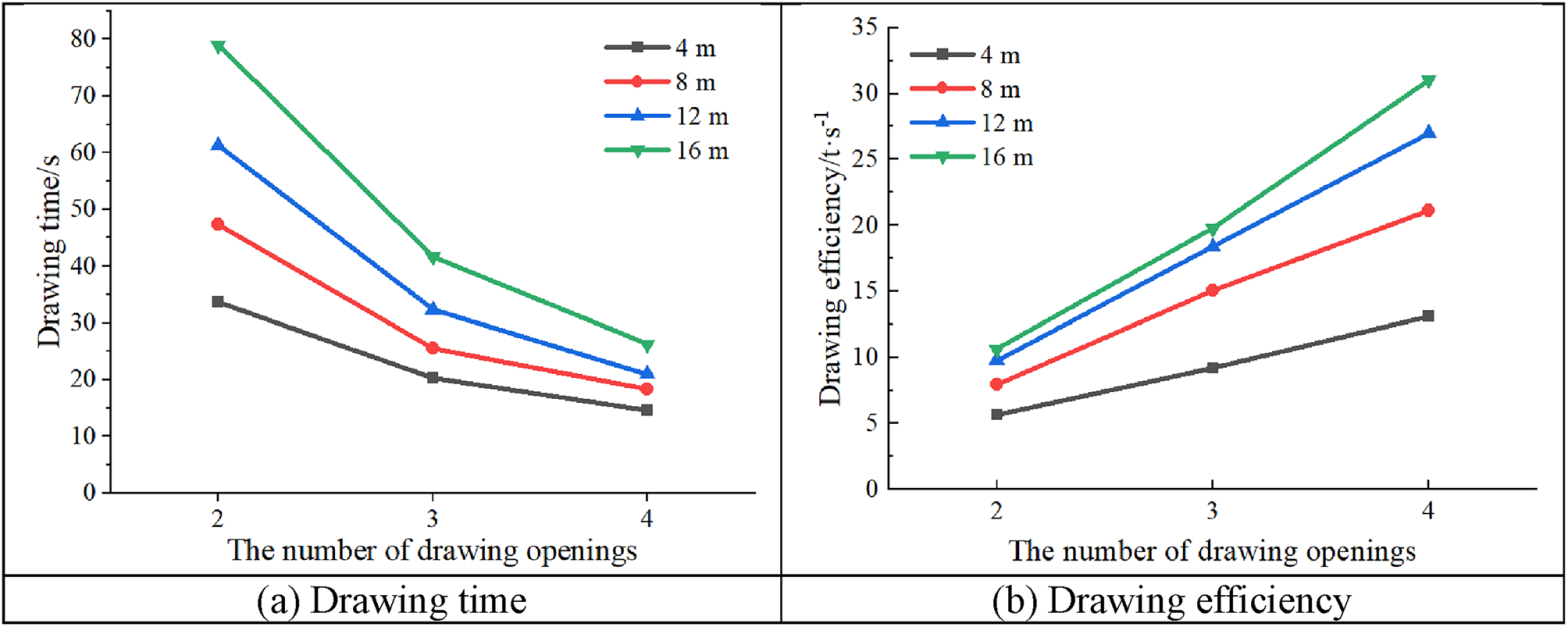
Time and efficiency under the single-round group drawing method.

Figure 10 shows the drawing time and efficiency for different top coal thicknesses. As shown in the figure, for the same top coal thickness, as the number of drawing openings increases, the drawing time gradually decreases and the drawing efficiency gradually increases. This is because increasing the number of drawing openings can reduce the number of simulated drawing times, thereby reducing the drawing time and increasing the drawing efficiency. Notably, when the number of drawing openings is greater than or equal to 2, the top coal no longer arches during drawing, which also reduces the drawing time to a certain extent. In addition, as the top coal thickness increases, the influence of the number of drawing openings on the drawing time and efficiency becomes more notable. Considering the top coal thicknesses of 4 and 16 m as examples, when the top coal thickness was 4 m, the number of drawing opens increased from 2 to 4, the drawing efficiency was 5.66%, 9.21% and 13.14% respectively, and the coal drawing time was 33.6 s, 20.3 s and 14.7 s respectively. Compared with the two-opening GDM, the three-opening GDM’s time reduced by 39.58% and efficiency increased by 62.72%, whereas the four-opening GDM’s time reduced by 56.25% and efficiency increased by 131.45%; when the top coal thickness was 16 m, the number of drawing opens increased from 2 to 4, the drawing efficiency was 10.66%, 19.78% and 31.04%, and the drawing time was 79.07 s, 41.65 s and 26.32 s, respectively. Compared with the two-opening GDM, the three-opening GDM’s time reduced by 47.32% and efficiency increased by 85.61%, whereas the four-opening GDM’s time reduced by 66.71% and efficiency increased by 191.20%. Notably, the two-dimensional model cannot correspond to the actual drawing time and efficiency on-site; the simulation results here are only used for horizontal trend comparison analysis.

### Top coal loss analysis

**Figure 11:**
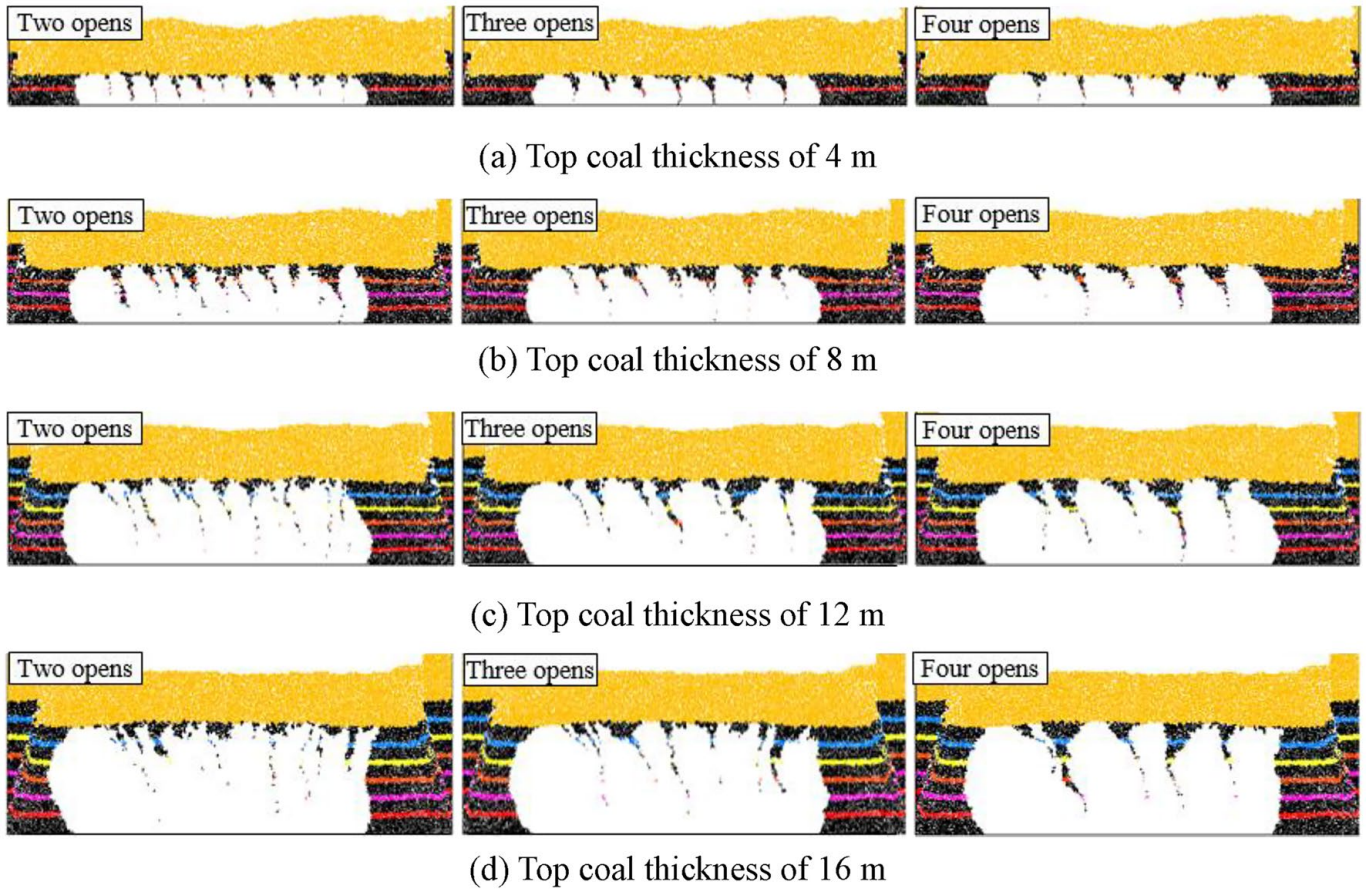
Original spatial position of the loss top coal under the single-round group drawing method.

Based on the above analysis, it can be concluded that TCR decreases with increasing top coal thickness. To investigate the top coal loss, the identification (ID) numbers of the top coal blocks drawn in each group of tests were extracted and recorded, and the blocks were deleted in the initial model. The blank area formed is the original spatial position of the top coal drawn, and the remaining top coal is the top coal that has not been drawn, which is the original spatial position of the lost top coal (Fig. 11).

As shown in Fig. 11, in the initial model, the shape of the lost top coal is roughly the shape of a “nail rake,” and the direction of the “rake teeth” is biased toward the direction of the model’s coal drawn (air roadway direction). The amount of top coal loss increases with the top coal layer, and the amount of top coal loss in the upper part is substantially greater than that in the lower part. In addition, for the same number of drawing openings, as the top coal thickness increases, the “rake teeth” shape becomes wider, indicating that an increase in the top coal thickness increases the top coal loss. As the number of coal drawing openings increases, the “rake teeth” become sparser, but the “rake teeth” volume gradually increases. This means that an increase in the number of drawing openings can, on the one hand, reduce the frequency of top coal loss, but, on the other hand, cause an increase in the amount of single top coal loss. That is, the impact of the number of drawing openings on the amount of top coal loss is influenced by both the drawing frequency of coal and the amount of single top coal loss, resulting in uncertainty in the direct impact of the number of drawing openings on the top coal loss.

(1) Analysis of top coal loss in different layers.

**Figure 12:**
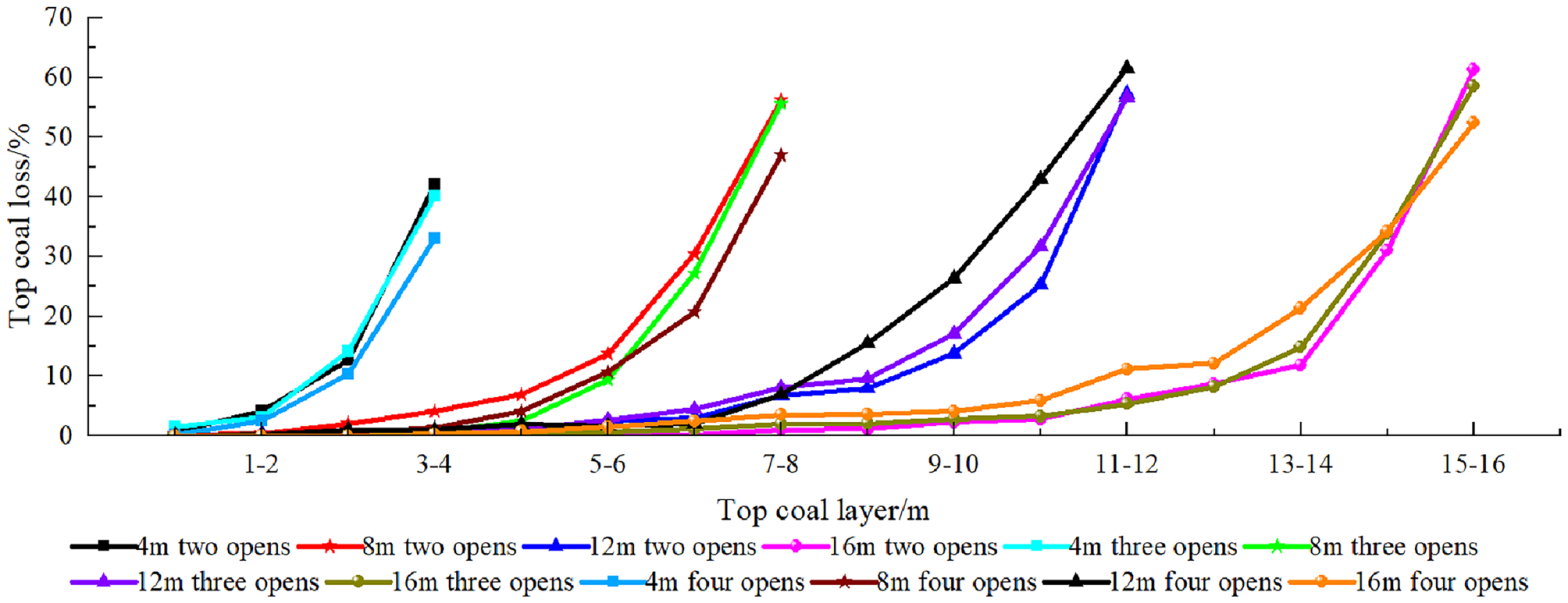
Loss rate of top coal at different layers under the single-round group drawing method.

To further analyze the top coal loss in different layers, a thickness layer of 1 m was used to calculate the top coal loss in each layer for different numbers of drawing openings and top coal thicknesses (Fig. 12). As the top coal thickness increases, the lower-top coal loss rate decreases, but the upper-top coal loss rate continues to increase. Considering the two-opening GDM as an example, while increasing the top coal thickness from 4 to 16 m, the top coal loss rate within the top-1-m range increased from 42.23 to 61.38%, indicating that the thicker the top coal, the more difficult it is to recover the upper coal. In addition, for top coal thicknesses of 4 and 8 m, the increase in the number of drawing openings decreases the top coal loss rate at different layers, and the higher the top coal layer, the greater the decrease in the loss rate. This indicates that the increase in the number of drawing openings is conducive to reducing the top coal loss rate, thereby achieving the goal of improving the TCR. However, for top coal thicknesses of 12 and 16 m, the top coal loss at different layers is exactly the opposite for different numbers of drawing openings.

In summary, there is a notable thickness effect of the number of drawing openings on the top coal loss. When the top coal thickness is ≤ 8 m, an increase in the number of drawing openings reduces the top coal loss in each layer. However, when the top coal thickness is > 8 m, an increase in the number of drawing openings increases the top coal loss in each layer. This is consistent with the relation between the TCR and the number of drawing openings under different top coal thicknesses.

(2) Impact mechanism of the number of drawing openings on top coal loss.

From the above analysis, the influence of the number of drawing openings on TCR has a top coal thickness effect. When the top coal thickness is small, the TCR increases with the number of drawing openings. When the top coal thickness is large, the TCR decreases with an increase in the number of drawing openings. To analyze the impact mechanism of the number of drawing openings on the top coal loss, considering the two- or four-opening GDM for top coal thicknesses of 4 and 16 m as examples, representative drawing times were selected to analyze the formation process of top coal loss. We extracted and recorded the ID numbers of the top coal blocks drawn for the nth time in each group of tests and deleted these blocks from the model after completion of the (n − 1)th coal drawing. The blank area formed is the top coal drawing body, DBn.

**Figure 13:**
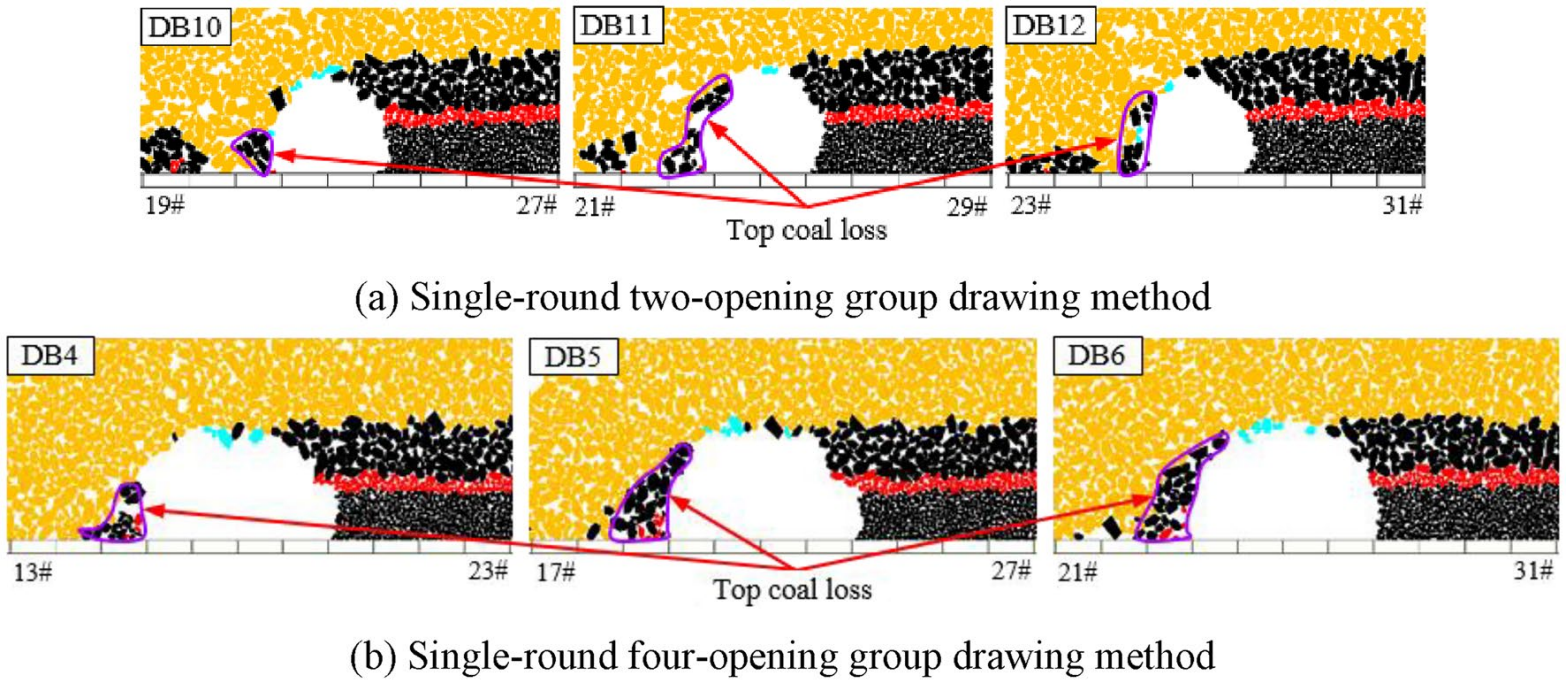
Top coal loss formation process under single-round group drawing method with a top coal thickness of 4 m.

Figure 13 shows the formation process of top coal loss for a single-round GDM with a top coal thickness of 4 m. When the top coal thickness is small, the distance between the coal–rock interface on the side of the drawing openings and the drawing openings is relatively small. The development height of the top coal drawing body is equal to the top coal thickness, and its lateral development degree reaches the limit level as the top coal thickness becomes constant. However, at the same development height, its lateral development degree gradually decreases with an increase in the number of drawing openings, and the top coal loss generated through single drawing also gradually increases. Nevertheless, an increase in the number of drawing openings can reduce the frequency of top coal loss, and its impact on the amount of top coal loss is dominant, thereby making the total amount of top coal loss gradually decrease with increasing number of drawing openings.

**Figure 14:**
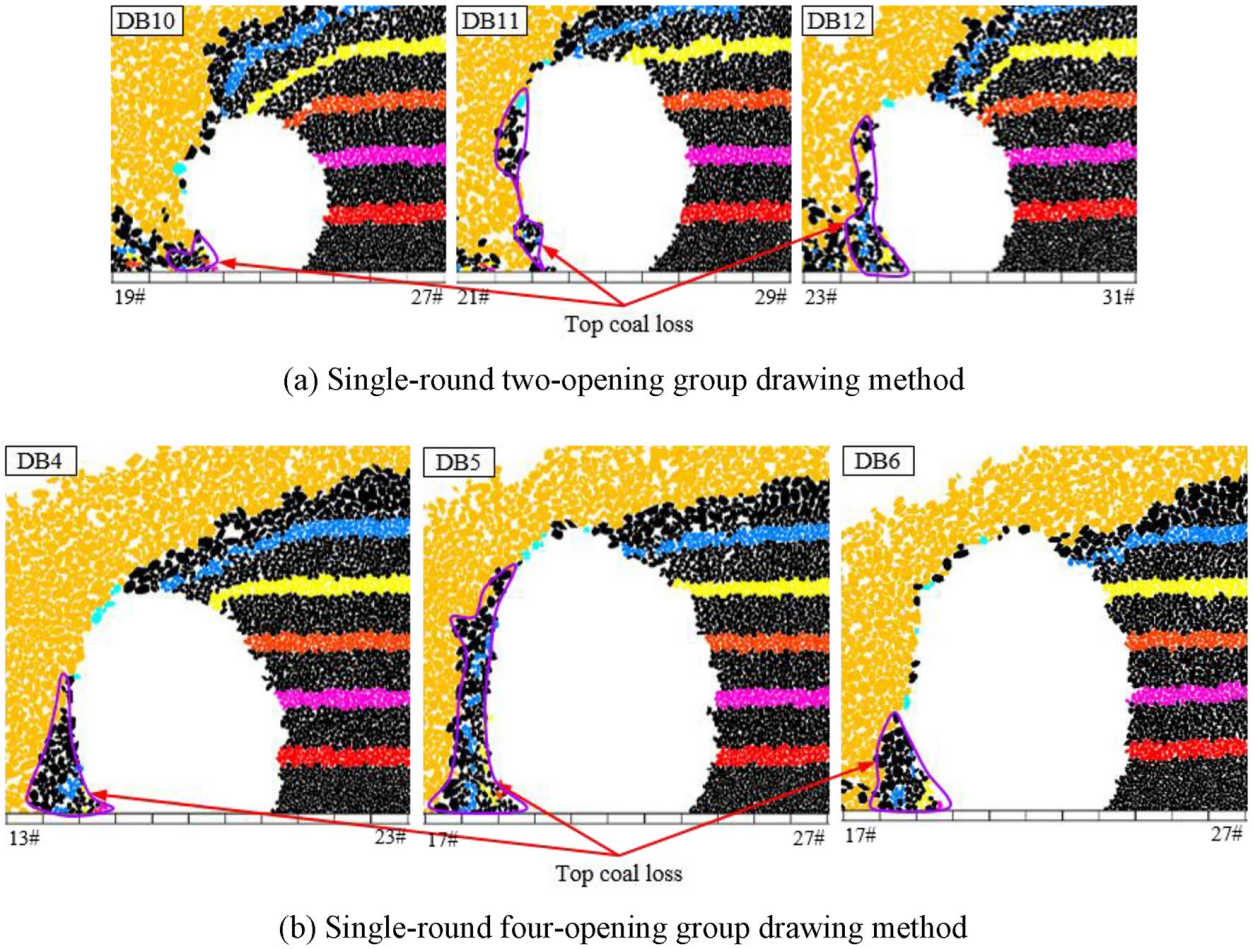
Top coal loss formation process under single-round group drawing method with a top coal thickness of 16 m.

Figure 14 shows the formation process of top coal loss for a single-round GDM with a top coal thickness of 16 m. When the top coal thickness is substantial, the distance between the coal–rock interface on the side of the drawing openings and the drawing openings is relatively far. The development height of the top coal drawing body is much lower than the top coal thickness, resulting in a low degree of lateral development. Accordingly, the top coal drawing body and coal–rock interface are mostly point-like and tangent, and numerous top coal that are difficult to draw below the tangent point exist, leading to a significant amount of top coal loss. As the number of drawing openings increases, the lateral development degree of the top coal drawing body continuously decreases, thereby considerably enhancing the top coal loss generated through a single drawing. This loss dominates the total loss of top coal, thereby gradually increasing the total amount of top coal loss with the increasing number of drawing openings.

### Relation between top coal recovery and gangue content

**Figure 15:**
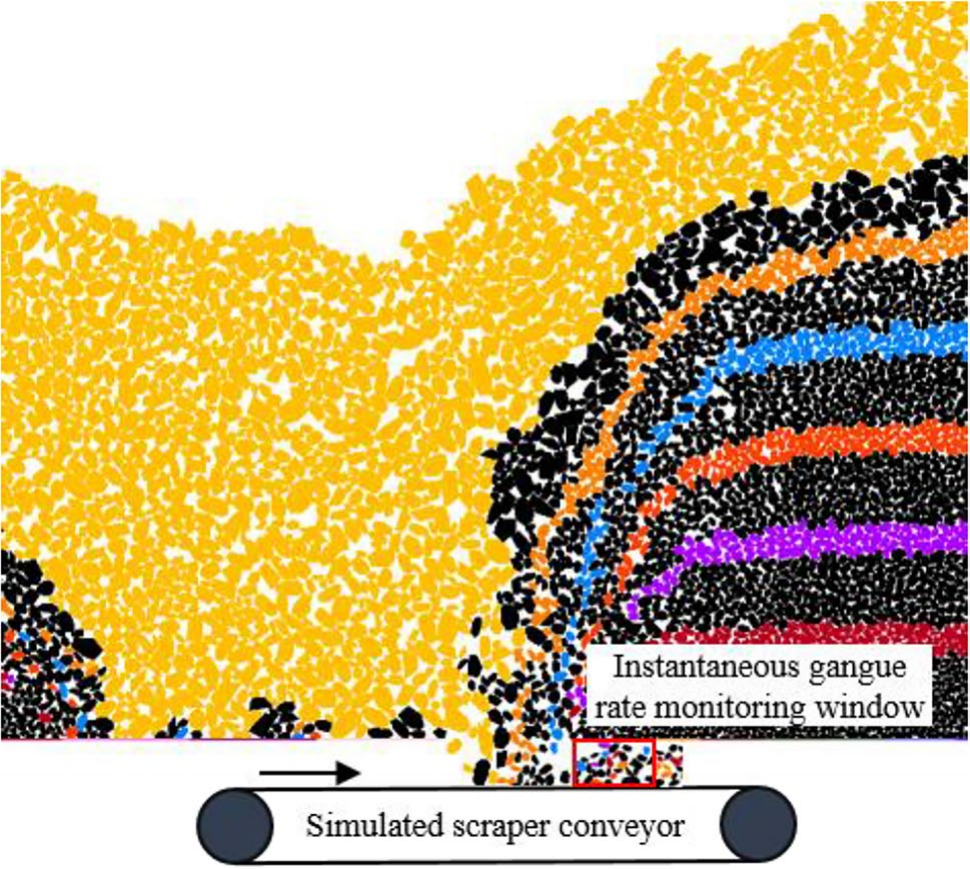
Schematic of instantaneous gangue content monitoring with support opening.

To reveal the relation between TCR and GC for single-round GDM, the IGC (volume proportion of gangue) at the drawing opening was calculated using a rectangular monitoring window during drawing (Fig. 15). When the IGC exceeds a set threshold, a “wall” structure element is instantly created at the drawing openings to simulate its closure. Notably, as the coal seam thickness increases, the range of immediate roof fragmentation rock block flow increases. However, an excessive drawing may result in the roof being very empty, which is not allowed in actual mining production. Accordingly, the maximum IGC thresholds in the top coal thickness schemes of 4, 8, 12, and 16 m were set to 80%, 70%, 60%, and 50%, respectively (Table 3).

**Table 3:** Experimental scheme of the group drawing method under different thresholds of instantaneous gangue content (IGC).

Scheme	Top coal thickness (m)	Number of drawing openings	IGC threshold (%)
1	4	2, 3, 4	10, 20, 30, 40, 50, 60, 70, 80
2	8		10, 20, 30, 40, 50, 60, 70
3	12		10, 20, 30, 40, 50, 60
4	16		10, 20, 30, 40, 50

**Figure 16:**
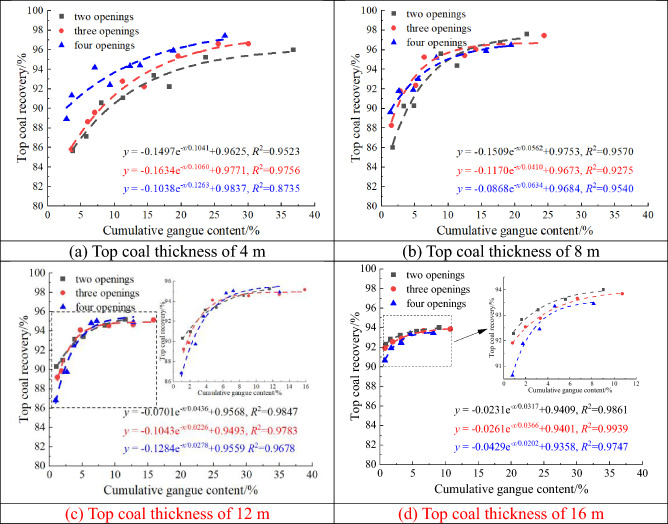
Relation between top coal recovery and cumulative gangue content for different top coal thicknesses.

CGC was obtained by calculating the proportion of gangue quality at the drawing openings. Figure 16 shows the relation between TCR with different thicknesses and CGC. TCR with GDM for different top coal thicknesses increases with CGC, but the trend gradually declines. TCR and CGC are correlated with a negative exponential relation (with a goodness of fit of > 0.87). When CGC is small, there is a considerable difference in the TCR values of the two-, three-, and four-opening GDMs. As CGC increases, the TCR values of the two-, three-, and four-opening GDMs gradually tend to be consistent, indicating that in the early stage of gangue formation, the coal drawn is mainly the top coal. By drawing an appropriate amount of gangue, TCR can be greatly improved. In the later stage of gangue formation, the amount of top coal drawn gradually decreases, whereas the amount of gangue gradually increases. At this time, it is difficult to recover more top coals by drawing the gangue. Moreover, TCR still increases with the number of drawing openings when the top coal thickness is 4 m. For a top coal thickness of 8 m, when CGC is low (< 13%), TCR increases with the number of drawing openings. However, when CGC is high (≥ 13%), the advantage of the TCR of the two-opening GDM begins to emerge. With the same CGC, the TCR of the two-opening GDM is greater than that of the three- or four-opening GDM. For top coal thicknesses of 12 and 16 m, TCR decreases with the increasing number of drawing openings. However, overall, for top coal thicknesses of 4 and 8 m, the TCR of the four-opening GDM is more advantageous (Fig. 16a,b), whereas, for top coal thicknesses of 12 and 16 m, the TCR of the two-opening GDM is the highest (Fig. 16c,d). This also indicates that when the top coal thickness is large (> 8 m), the fewer drawing openings opened simultaneously, the better the effect of the top coal drawn.

In addition, as shown in Fig. 16, with an increase in top coal thickness, the effect of increasing CGC on the TCR rate gradually weakens, and the limit value of TCR gradually decreases with increasing top coal thickness. This means that as the top coal thickness increases, the effect of increasing CGC on the TCR improvement is limited; the TCR improvement has already been completed when CGC is small. Therefore, when the top coal thickness is large, a relatively good TCR can be achieved with a lower CGC.

**Figure 17:**
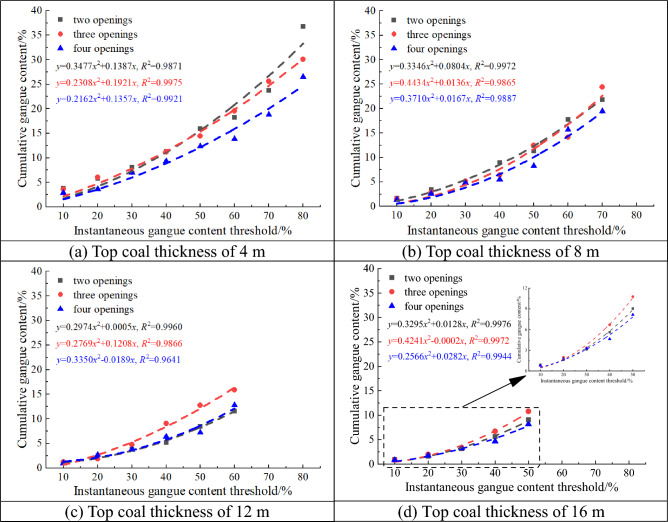
Relation between cumulative gangue content and instantaneous gangue content threshold for different top coal thicknesses.

Figure 17 shows the relation between the CGC and IGC thresholds for different top coal thicknesses. CGC increases with the IGC threshold, and the growth rate continues to increase. They have a quadratic relation, with a goodness of fit of > 0.96, indicating that it is completely feasible to use the IGC threshold at the drawing openings to infer CGC, which also provides a basis for determining the timing of closing the openings in the working face. In addition, when the IGC threshold is small, there is not much difference in CGC among the two-, three-, and four-opening GDMs. However, as the IGC threshold increases, the difference in CGC among them gradually increases. This is because the IGC at the opening is the proportion of gangue volume, but CGC is the proportion of mass, and the coal gangue density substantially varies (gangue density can reach approximately 1.8 times the coal density). Therefore, as the IGC threshold increases, the CGC growth rate increases.

In addition, as shown in Fig. 17, as the top coal thickness gradually increases, the CGC growth rate continues to de-accelerate. With the same IGC threshold, the thicker the top coal, the lower the CGC, and the lower the coal washing or sorting costs caused by the appropriate gangue drawing. This is beneficial for some fully mechanized working faces with thick top coal. Considering the two-opening GDM as an example, at an IGC threshold of 30%, CGC decreased from 7.07 to 3.33% when the top coal thickness was increased from 4 to 16 m.

In summary, for top coal thicknesses of 4 and 8 m, it is advisable to use the four-opening group drawing technology. Meanwhile, for top coal thicknesses of 12 and 16 m, it is advisable to use the two-opening group drawing technology. Without considering the increase in coal washing or sorting costs caused by excessive coal drawing, from the perspective of improving TCR, with a growth rate of less than 10% as the boundary, CGC should be greater than 21% and 14%, respectively, for top coal thicknesses of 4 and 8 m, and greater than 11% and 6%, respectively, for top coal thicknesses of 12 and 16 m. From the perspective of setting the IGC threshold at the drawing opening, for top coal thicknesses of 4 and 8 m, the IGC threshold for the four-opening GDM should be set to approximately 70% and 60%, respectively; meanwhile, for top coal thicknesses of 12 and 16 m, the IGC threshold for the two-opening GDM should be set to approximately 50% and 40%, respectively.

According to the theoretical analysis, it can be seen that there is a quantitative relationship between the CGC, the IGC and the TCR, but the theoretical analysis only gives the specific expression of the IGC and the CGC. To establish the quantitative relationship between the GC and the TCR, this paper uses the numerical simulation method to fit the relationship curve between the CGC and the TCR under the two-opening GDM. It is found that when the CGC is about 10%, the TCR no longer increases with the increase of the CGC. Then, the CGC of 10% is brought into the theoretical analysis to obtain the relationship between the IGC and the CGC, and the theoretical value of the IGC corresponding to the coal drawing openings is 31.2%. Therefore, the integrated utilization of theoretical analysis and numerical simulation has led to the determination that the reasonable threshold of IGC at the coal drawing openings of the 8222 working face in Tashan Mine lies between 31.2 and 40%.

## Design and on-site application of group drawing method

### Drawing process design

The 8222 working face of the Tashan Coal Mine is the first intelligent fully mechanized working face of the Jinneng Coal Industry Group, with an annual output of 15 million tons of the extra-thick coal seam. The working face began to be mined in January 2019, and after the initial mining stage, the working face production gradually stabilized. In the early stages of mining at the working face (March and April 2019), the “four-level” coal drawing method was adopted, with an average TCR of approximately 75.25%^31^. To improve the drawing efficiency and TCR, the drawing method of the 8222 working face was optimized and adjusted from the “four-level” coal drawing method to GDM. Because the automation and intelligent control of the drawing process is still in the experimental stage, substantial manual intervention is required. Considering that the production capacity target of the 8222 working faces is 15 million tons/a, a single-round GDM with higher drawing efficiency was adopted during the industrial trial period. Considering various factors, such as the top coal thickness, gas, dust, and conveying capacity of the scraper conveyor associated with the working face, the number of simultaneous drawing openings was ultimately determined to be two. To achieve the highest economic benefits of mining and sorting, the IGC threshold at the drawing openings was set to 35% (openings should be immediately closed when the volume of gangue reaches approximately 1/3). To accurately monitor the IGC at the openings, hyperspectral and coal gangue impact–vibration–signal recognition technologies were used to identify the coal gangue at the drawing openings during drawing^32^. When the IGC reached 35%, the drawing openings were closed. During mining, the drawing amount was measured in real time using a belt scale arranged at the machine roadway to calculate TCR.

### Benefit analysis of the on-site application

From January 16 to April 15, 2020, the cumulative advance of the working face was 672 m and the production of raw coal was ~ 4.042 million tons, with an average monthly output of 1.347 million tons. The average recovery rate of the working face was 92.0%, and the average GC was 9.25% (Table 4). By accurately controlling the IGC at the drawing openings, the working face TCR was increased to 90.12% and the GC was controlled at ~ 9%, which is ~ 14.87% higher than that of the “four-level” coal drawing method. In addition, by increasing the space size of the drawing openings, the GDM greatly improved the working face production efficiency and reduced the frequency of top coal arching and blocking the drawing openings. During industrial tests, the average work efficiency reached 400 ton work^−1^, the average drawing efficiency was 2954 ton h^−1^, and the coordination efficiency of mining and drawing time reached 68.2%.

**Table 4:** Production conditions of the working face during industrial tests.

Month	Monthly production (t)	Advance distance (m)	Average daily production (t)	Average work efficiency ton work^−1^	Working face TCR (%)	GC (%)
1.16–2.15	1,362,756	223.6	45,425.2	405	94.12	9.86
2.16–3.15	1,305,681	219.8	45,023.5	399	88.9	8.55
3.16–4.15	1,373,562	228.6	45,785.4	397	92.9	9.33
Average	1,347,333	–	45,411.4	400	92.0	9.25

The production data during the industrial tests show that the 8222 intelligent fully mechanized working face had an annual production capacity of 15 million tons. The application of the single-round GDM provided key technical support for fully mechanized drawing mining of extra-thick coal seams, which is important for the high-yield and efficient development of such seams.

## Conclusions

1. Based on the B–R model, the evolution equation of the top coal drawing body and the coal-rock interface under the GDM is further deduced, and the quantitative relationship between the IGC and the CGC is obtained. The feasibility of using the IGC to predict the CGC and the TCR is demonstrated.
2. As the top coal thickness increases, there is a notable thickness effect of the number of drawing openings on TCR. When the top coal thickness are 4 and 8 m, the TCR gradually increases with an increase in the number of drawing openings, whereas for top coal thicknesses of 12 and 16 m, the TCR gradually decreases with an increase in the number of drawing openings. And as the top coal thickness increases, the influence of the number of coal drawing openings on the coal drawing time and efficiency becomes more significant.
3. TCR increases with CGC, but the growth trend gradually decelerates; they have a negative exponential relation. Moreover, as the top coal thickness increases, the growth effect of CGC on TCR gradually weakens, and the limit value of TCR gradually decreases with increasing top coal thickness. CGC increases with the IGC threshold, and the growth rate continues to increase. They exhibit a quadratic relation. The thicker the top coal, the lower the CGC, and the lower the increase in coal washing or sorting costs caused by the appropriate gangue drawing. This is beneficial for fully mechanized working faces with high top coal thicknesses.
4. From the perspective of TCR improvement, the 4 m and 8 m thick coal seams should use the four-opening GDM, CGC should be greater than 21% and 14%, respectively, and the IGC threshold should be set to approximately 70% and 60%, respectively. For top coal thicknesses of 12 and 16 m, it is advisable to use the two-opening GDM, with CGC greater than 11% and 6%, respectively, and the IGC threshold should be set to approximately 50% and 40%, respectively.
5. Considering various factors, such as the top coal thickness, gas, dust, and conveying capacity of the scraper conveyor associated with the working face and economic benefits of mining and sorting, the process parameters of group drawing in working face are designed, which is a two-opening GDM with an IGC threshold of 35% at the coal drawing openings. The industrial test results after optimizing and adjusting the coal drawing technology show that the working face TCR increased from 75.25 to 90.12%, a 14.87% increase, and the average CGC was 9.25%. In addition, the average coordination efficiency of coal mining and drawing time could reach 68.2%.

## Data Availability

The datasets generated and/or analyzed during the current study are available from the corresponding author upon reasonable request.
